# A Color-Detectable Vitamin C Controlled-Release System Fabricated Using Electrospinning

**DOI:** 10.3390/polym16101347

**Published:** 2024-05-09

**Authors:** Min Jae Shin

**Affiliations:** Department of Chemical and Biological Engineering, Andong National University, Andong 36729, Gyeongbuk, Republic of Korea; newminj@gmail.com

**Keywords:** vitamin C, controlled-release system, electrospinning, polydiacetylene

## Abstract

This study develops a vitamin C controlled-release system, trackable via color changes as a function of vitamin C release. The system is composed of coaxial microfibers prepared via coaxial electrospinning, with a core of poly(ethylene oxide) (PEO) incorporating vitamin C, and a shell composed of polycaprolactone (PCL) containing polydiacetylene (PDA) as the color-changing material. The shell thickness is controlled by adjusting the amount of PCL ejected during electrospinning, allowing regulation of the release rate of vitamin C. When vitamin C added to PEO penetrates the PCL layer, the color of PDA changes from blue to red, indicating a color change. The results of this study can be applied to devices that require immediate detection of vitamin C release levels.

## 1. Introduction

Vitamin C, also known as L-ascorbic acid, is a water-soluble vitamin that plays a crucial role in the development of various organs in the body. It is involved in collagen synthesis and skin formation, and it acts as an antioxidant by preventing the oxidation of various substances within the body. However, vitamin C is prone to oxidation, owing to its antioxidant properties. Therefore, research and applications involving vitamin C require significant effort to stabilize it and prevent its degradation [1,2,3].

Encapsulation using polymeric materials is commonly employed for stabilizing unstable organic substances [4,5,6]. Well known techniques such as solvent evaporation, spray drying, and electrospinning have been widely used for this purpose. In solvent evaporation, the target organic substance solution is dispersed in a larger volume of water by intense stirring, and the polymer solution is slowly added dropwise. During this process, the organic solvent evaporates, encapsulating the organic substances within the polymer matrix. Although this method provides clean encapsulation, it presents challenges when handling substances that completely dissolve in water, making it less suitable for water-soluble compounds such as vitamin C [7,8,9,10].

Spray drying involves spraying an aqueous solution containing the target organic substance and the polymer material into air. Encapsulation occurs when the solvent evaporates. Although this method allows the encapsulation of water-soluble organic substances, the resulting microcapsules often possess rough surfaces, leading to inferior encapsulation compared with the solvent evaporation method [11,12].

Electrospinning produces microfibers by subjecting a polymer solution to a high-voltage electric field. Encapsulation can be achieved by incorporating a target organic substance into a polymer solution. The fibers formed by electrospinning have diameters ranging from tens of nanometers to several micrometers, resulting in structures with large surface areas. Consequently, when an electrospun microfiber is used in a controlled release experiment, an initial burst-release phenomenon occurs, which is characterized by a significant release of the encapsulated substance during the initial stages. This phenomenon has been observed in various organic substances and polymers [13,14].

Traditional electrospinning involves the fabrication of fibers from a single polymer solution or melt. However, the development of coaxial electrospinning has enabled the encapsulation of active pharmaceutical ingredients within core-shell structured fibers, offering enhanced control over drug release kinetics. The coaxial configuration allows for the independent optimization of the core and shell materials, facilitating tailored release profiles to meet specific therapeutic requirements. In recent years, coaxial electrospinning has garnered considerable attention for the fabrication of controlled release systems, owing to its unique advantages. By modulating the properties of the core and shell materials, researchers can achieve diverse release kinetics, including sustained, pulsatile, or triggered release, thereby expanding the scope of applications across various therapeutic areas. Furthermore, the scalability and reproducibility of coaxial electrospinning make it a promising platform for the development of commercial drug delivery products. To achieve complete encapsulation, this study employs a coaxial electrospinning system, in which the encapsulating material forms the outer shell of the coaxial structure. By controlling the amount of polymer material ejected during electrospinning, the thickness of the outer shell can be adjusted, enabling the regulation of the release rate of the encapsulated substance [15,16,17,18,19].

Polydiacetylene (PDA) is a conjugated polymer in which double and triple bonds are alternately conjugated. PDA exhibits color changes in response to external stimuli, making it suitable for various sensor applications. Previous studies have reported the development of sensor systems using PDA [20,21,22,23,24].

This study aimed to manufacture a system that incorporates PDA into the outer part of a coaxial electrospinning system. By modulating the release of vitamin C, this system exhibits a color change from blue to red, enabling visual tracking of the release process. This study focused on developing a visually trackable vitamin C controlled-release system. In this study, two methods were attempted to induce a color change through the release of vitamin C. Firstly, using vesicles formed by PDA, we observed the color change as vitamin C permeated from the outside to the inside of the vesicle. Secondly, and crucially to this study, we fabricated a controlled release system using coaxial electrospinning. Here, vitamin C encapsulated within the core fiber releases outward while exhibiting a color change due to PDA present in the shell fiber. Coaxial electrospinning was employed to fabricate coaxial microfibers in which poly(ethylene oxide) (PEO) and vitamin C formed the core fiber, and polycaprolactone (PCL) with PDA formed the shell fiber. The release rate of vitamin C was regulated by adjusting the amount of PCL injected during electrospinning. The color change from blue to red, resulting from the passage of vitamin C through the PCL layer, can be utilized in devices requiring immediate detection of vitamin C penetration.

## 2. Experimental

### 2.1. Materials and Equipment

PCL (Mn 80,000), 10,12-pentacosadiynoic acid (PCDA), PEO (Mv 900,000), L-ascorbic acid, *N*-hydroxysuccinimide (NHS), *N*-(3-dimethylaminopropyl)-*N’*-ethylcarbodiimide (EDC), ethylenediamine, dimethylformamide (DMF), chloroform, and dichloromethane (DCM) were purchased from Sigma-Aldrich (St. Louis, MO, USA). The ultraviolet (UV) spectra were obtained using a Shimadzu UV-1800 spectrophotometer (Kyoto, Japan). The ^1^H NMR spectra were obtained using a Bruker DRX 300 spectrometer (Billerica, MA, USA). The size of the vesicles was measured using ELSZ-2 dynamic light scattering (DLS) (Photal Otsuka Electronics, Osaka, Japan). The coaxial fibers were fabricated using NanoNC ESR 100D electrospinning equipment (Seoul, Republic of Korea), and the electrospun fiber mats were collected on a NanoNC-DC90H drum-type collector (Seoul, Republic of Korea) with a 94.5 mm diameter. The coaxial nozzle specifications for the electrospinning process are listed in Table 1. The fibers were imaged using an Olympus BX53MRF-S optical microscope (Tokyo, Japan). The dialysis bag purchased from Sigma-Aldrich had a molecular weight cut-off of 14,000.

### 2.2. Synthesis of N-(2-Aminoethyl)pentacosa-10,12-diynamide (AEPCDA)

AEPCDA was synthesized as described in previous publications [25,26,27]. PCDA (3.75 g, 10.0 mmol), EDC (2.88 g, 15.0 mmol), and NHS (1.50 g, 13.0 mmol) were agitated in 20 mL of DCM at 30 °C for 3 h to synthesize PCDA-NHS. DCM was evaporated under a vacuum, and the residue was purified by extraction with ethyl acetate. PCDA-NHS (3.73 g, 7.91 mmol) was obtained as a white solid (yield: 79.1%). PCDA-NHS (2.50 g, 5.29 mmol), in 50 mL of DCM, was added very slowly to ethylenediamine (1.00 g, 16.6 mmol) in 100 mL of DCM to synthesize the AEPCDA. The reaction continued for 6 h at 30 °C, then the precipitate was filtered out and DCM in the residual filtrate was evaporated under vacuum. The product was extracted from the residue using DCM, and the solution was washed with brine twice. The product was purified via recrystallization with DCM. AEPCDA (1.21 g, 2.91 mmol) was obtained as a white solid (yield: 55.0%).

^1^H NMR (CDCl_3_): 0.90 (t, 3H), 1.28 (s, 26 H), 1.45–1.54 (m, 6H), 2.15 (t, 2H), 2.25 (t, 4H), 2.81 (m, 2H), 3.37 (m, 2H), 4.58 (s, 2H), 6.45 (s, 1H).

### 2.3. Formation of PDA Vesicle Solution

AEPCDA (20.8 mg, 0.500 × 10^−4^ mol) was dissolved in 10 mL of chloroform in a 100 mL round-bottom flask. Chloroform was evaporated under a vacuum, leaving AEPCDA as a thin membrane at the bottom of the flask. After completely evaporating chloroform, 50 mL of water was added to the flask. The mixture was sonicated at 70 °C for 30 min to prepare a 1.00 mM AEPCDA vesicle solution. AEPCDA was photopolymerized by transferring the solution to a Petri dish and subjecting it to UV irradiation (254 nm) for 30 s.

### 2.4. Electrospinning

Electrospinning was performed using a coaxial nozzle. The applied voltage was 14.0 kV, and the distance between the nozzle and the collector was 15.0 cm. The solution for the core formation was prepared as follows: 0.350 g of PEO was dissolved with rapid stirring (1500 rpm), and 150 mg of vitamin C was added. After evaporating the solution in a rotary evaporator, 10.0 mL of CHCl_3_ was added to the flask and processed for 15 h in a bath sonicator to produce the core-forming solution. The shell-forming solution was prepared by adding 1.20 g of PCL to a mixture of 8.0 mL DCM and 2.0 mL DMF as solvent, and rapidly stirring the mixture (1500 rpm). To observe the color change using PDA, 20 mg of AEPCDA was added to the PCL solution in five 4.0 mg portions during the preparation of the shell-forming solution.

### 2.5. Vitamin C Release

The vitamin C release experiment used a mat composed of electrospun fibers with a PEO core containing vitamin C and a PCL shell. Initially, electrospinning was conducted for 1 h to prepare the fibers, which were then collected and placed in a dialysis bag. The release experiment was performed in 1000 mL of distilled water. The amount of released vitamin C was measured by determining the absorbance at 266 nm in the UV spectrum.

## 3. Results and Discussion

**Figure 1:**
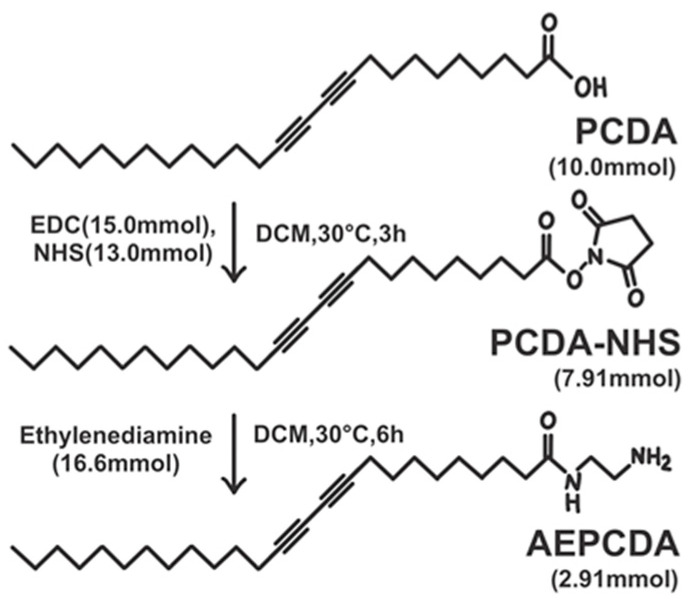
AEPCDA synthesis procedure.

Commercially available PCDA is the most commonly used diacetylene compound in sensing research utilizing PDA. It has a diacetylene group in the middle and a carboxylic acid functional group at the end, with a carbon chain length of 25. Prior to entering this study, PCDA was sonicated in water to form a vesicle solution. However, when this solution was used to detect vitamin C, no color changes were observed. Therefore, a synthesis experiment was conducted to convert the carboxylic acid functional group at the end of PCDA into an amine group. PCDA-NHS was synthesized by reacting PCDA with EDC and NHS. Subsequently, AEPCDA was synthesized by reacting the thus formed PCDA-NHS with ethylenediamine. This process is illustrated in Figure 1.

### 3.1. Vitamin C Detection Using AEPCDA Vesicle Solution

**Figure 2:**
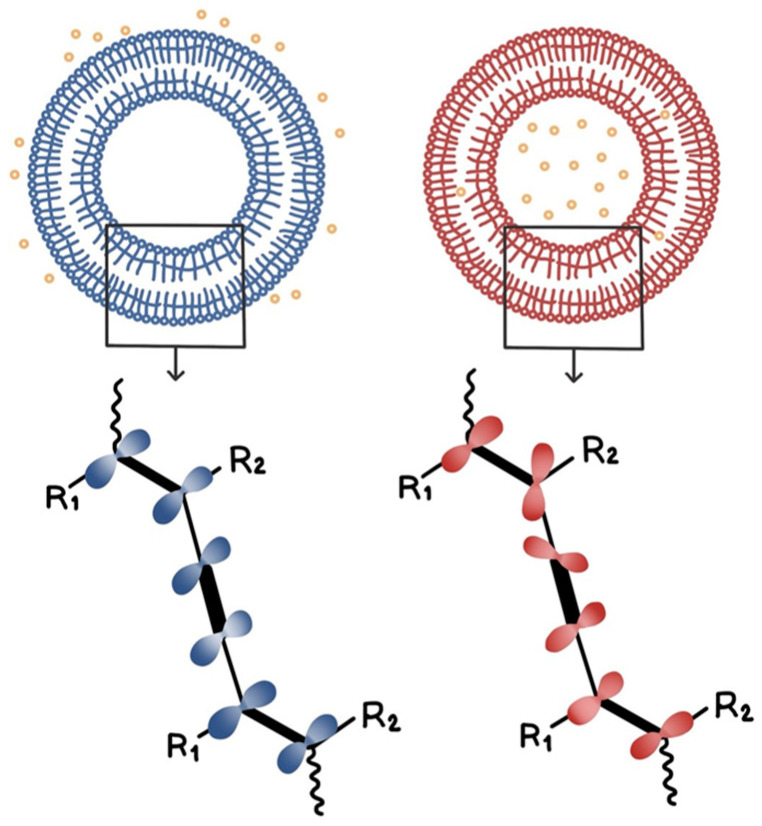
The change in vesicle color from blue to red upon vitamin C penetration from the outside to the inside of the vesicle.

**Figure 3:**
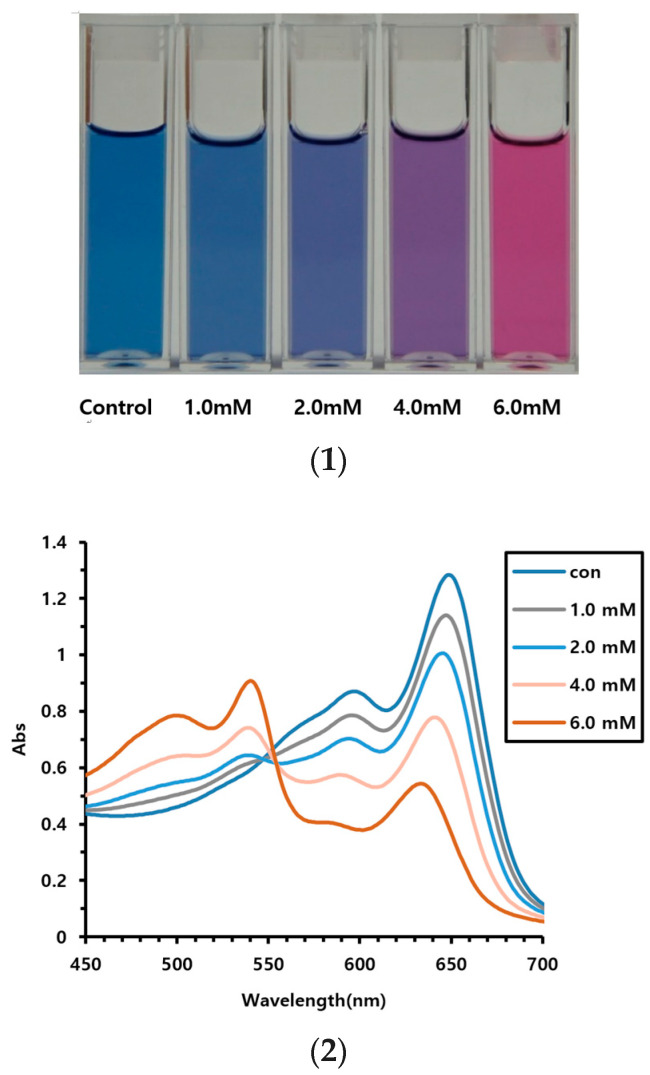
Vitamin C detection using AEPCDA vesicle solution. (**1**) Photographs of color change, (**2**) UV spectrum of the vesicle solution.

First, the ability to detect vitamin C using AEPCDA synthesized in this study was assessed using the AEPCDA vesicle solution. The vesicle solution was prepared by sonication and polymerized using UV irradiation (254 nm). The size of the produced polymerized vesicles was measured using DLS, with an average diameter of 220 ± 30 nm. When the polymerized vesicles were prepared using AEPCDA, a color change from blue to red was observed as vitamin C penetrated from the external environment to the inside of the vesicle, as shown in Figure 2. The results of this experiment are shown in Figure 3.

From the image in Figure 3, it can be observed that color changes can be detected from a vitamin C concentration of 2.0 mM. At 4.0 mM, the color changed to purple, while at 6.0 mM, a complete color change to red was observed. Furthermore, in terms of UV spectra, the blue UV absorption between 630 and 650 nm decreased with increasing vitamin C concentration, while the red absorption near 540 nm gradually increased.

No color changes were detected in our preliminary experiment using PCDA. Conversely, color changes from blue to red were detected using AEPCDA. This was presumably induced by the strong interactions between the acid functional group of vitamin C molecules and the amine group at the end of AEPCDA.

In this study, when sonication was applied to diacetylene compounds, vesicles were formed, and at this point, the diacetylene groups aligned parallel to each other, enabling polymerization under UV light. This polymerization process occurring in this manner is referred to as topochemical polymerization. Through this process, PDA was formed. PDA exhibits a blue coloration due to the alternating arrangement of double and triple bonds in its conjugated unsaturated bonds.

The mechanism behind the color change in PDA from blue to red in response to stimuli has been the subject of numerous studies since the phenomenon was discovered, but it has not been fully elucidated with certainty. It is currently understood that the color of PDA is determined by the length of conjugation of the conjugated bonds lying within a single plane, which is known to be related to the interactions between hydrophilic head groups. Therefore, it is known that the color can change in response to external stimuli that affect the interaction of these hydrophilic head groups. However, the exact reason for the shift from blue to red is still not definitively understood.

### 3.2. Detecting Vitamin C Release from Microfiber Mat

In this experiment, vitamin C release was detected using a mat of coaxially electrospun fibers with a vitamin C-containing PEO core and an outer shell of PCL containing PDA as the sensing material undergoing the color change. Thus, when vitamin C in the inner part was released into the outer part (PCL) of the fiber, it encounters PDA, resulting in the color change.

**Figure 4:**
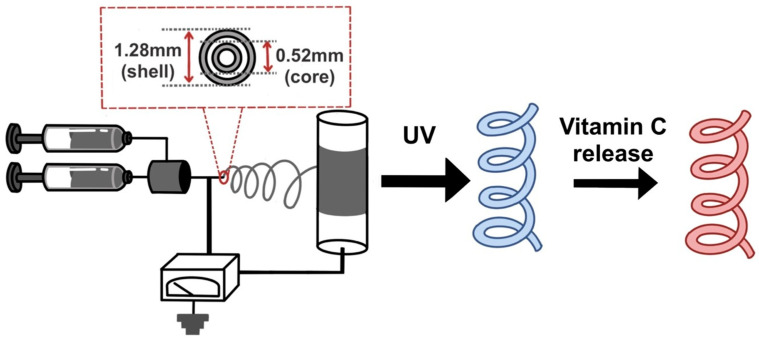
Electrospinning device and coaxial nozzle, along with the experimental process.

Figure 4 shows the electrospinning device with a coaxial nozzle, and the experimental process used in this study.

Three coaxial microfiber types were used in this experiment, and the electrospinning conditions and fiber thicknesses are summarized in Table 2. In this experiment, we only presented the results of varying the flow rate as it pertained to changes in the morphology of the microfibers formed during electrospinning. It is well known that there are several experimental factors that can influence the morphology of microfibers formed during electrospinning experiments. These factors include variations in applied voltage, the distance between the syringe needle and the collector, the solvent used in the polymer solution, and the concentration of the polymer solution, among others. While we attempted experiments by varying most of these factors, the primary focus of this study was to achieve clear formation of coaxial fibers and vary the thickness of the shell fiber. Therefore, we kept most of the factors that led to optimal coaxial fiber formation fixed, and solely varied the flow rate of the polymer solution for shell formation, as it was the factor that best allowed for variation in shell thickness.

**Figure 5:**
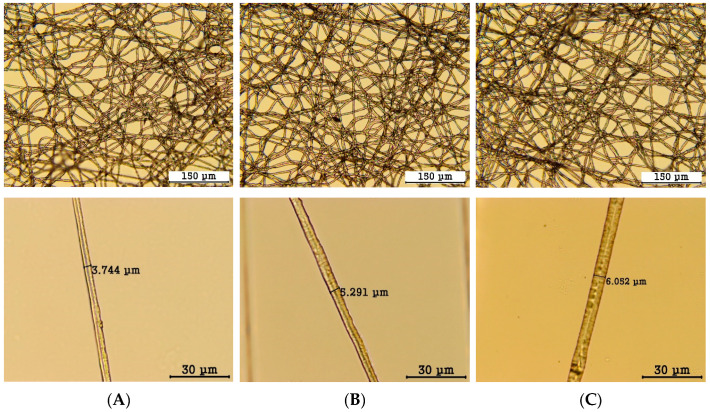
Microscope images of the fibers. (**A**–**C**) refer to the same variables as in Table 2.

The morphology of the formed fibers was observed using a microscope at magnifications of 200× and 1000×, and the results are shown in Figure 5.

The average fiber diameters obtained from Figure 5 for A, B, and C are 3.9 ± 0.6 μm, 4.8 ± 0.6 μm, and 5.7 ± 0.7 μm, respectively, indicating that increasing the flow rate of the shell portion of the coaxial spinneret resulted in an increase in the size of the shell portion, leading to an overall increase in the fiber diameter.

**Figure 6:**
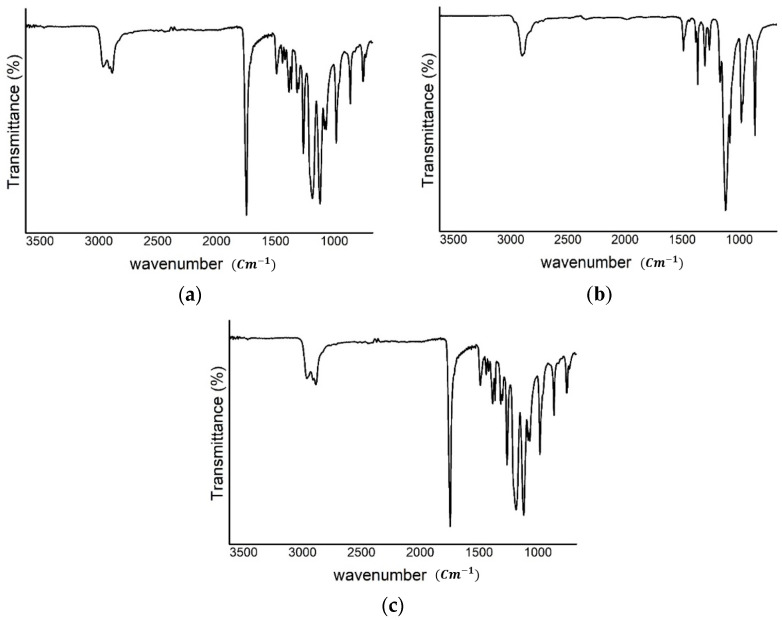
IR spectrum of (**a**) PCL, (**b**) PEO, and (**c**) electrospun fiber with PCL/PEO.

The IR spectra of the fibers were obtained from the samples shown in Table 2 (B) and compared with the IR spectra obtained from the basic materials of the fibers: PEO and PCL. The results are shown in Figure 6.

The IR spectrum of PCL in Figure 6a includes absorption peaks at 1723 cm^−1^ and 1164 cm^−1^, indicating absorption by the carbonyl and C–O groups, respectively. The IR spectrum of PEO in Figure 6b shows an absorption peak at 1098 cm^−1^, indicating absorption by the C–O group. These absorption peaks are all visible in the IR spectrum of the electrospun fiber with PCL/PEO (Figure 6c), indicating that the fiber was composed of both PCL and PEO.

**Figure 7:**
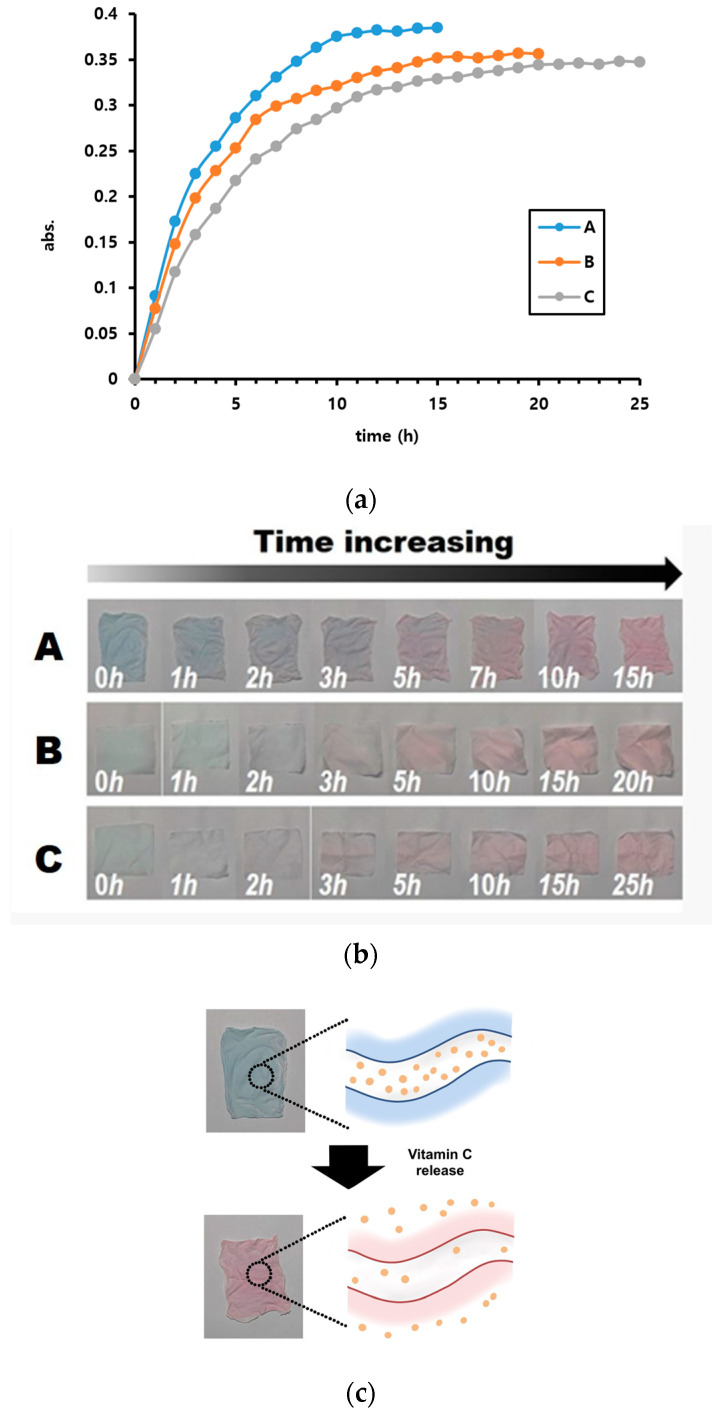
(**a**) Changes in the concentration of released vitamin C with time. A, B, and C refer to the same variables as in Table 2. (**b**) Color change in the fiber-mat due to the increase in the amount of penetrating vitamin C. A, B, and C refer to the same variables as in Table 2. (**c**) The process of color change occurring.

The release experiments were conducted, and the concentrations of released vitamin C over time are plotted in Figure 7. Additionally, the color changes resulting from the release of vitamin C at each point are shown in Figure 7.

From Figure 7, it is evident that the release rate can be controlled by varying the shell thickness. In case A, nearly all the releasable vitamin C was released after 10–11 h. In contrast, this occurred after 15–16 h and 21–22 h in cases B and C respectively. Additionally, the final amount of released vitamin C differed slightly, in the order of A > B > C. This was attributed to the presence of captured vitamin C that was difficult to release, with quantities of captured vitamin C being in the order C > B > A. Furthermore, the color changes observed at each point in Figure 7b show that the color changed from blue to red as time progressed.

We conducted the vitamin C release experiment using coaxial microfibers fabricated once (Figure 7a), and repeated it five times. The error in the measured UV values during these experiments was ±7%. Among these five experiments, we display the results of the second experiment, which showed a moderate value, in Figure 7a.

Subsequently, we conducted vitamin C release experiments using newly fabricated coaxial microfibers under the same conditions, repeating the experiment twice for a total of three trials. The error in the measured UV values during these experiments was ±15%, showing a relatively large difference. However, whether using the coaxial microfibers fabricated once or newly, the slopes of the graphs in panels a, b, and c varied proportionally, resulting in overall similar graph shapes. Therefore, these differences did not significantly impact the interpretation of the experimental results.

In the experiments conducted with the vesicles mentioned earlier, the process of forming blue-colored PDA through topochemical polymerization by aligning diacetylene compounds in electrospun microfiber mats was also carried out. The main difference is that in the experiments using vesicles, sonication was used to align the diacetylene compounds, whereas the diacetylene compounds added to PCL arranged themselves within the polymer matrix of PCL. In this arranged state, upon exposure to UV light, topochemical polymerization occurred, leading to the formation of PDA, and consequently, the color of the mat changed from colorless to blue. Additionally, when vitamin C contained in the core portion was released into the shell portion, contact occurred between the PDA and vitamin C in the shell portion, leading to a color change in PDA from blue to red.

In conclusion, this study successfully fabricated a controlled release system using coaxial electrospinning, resulting in color change triggered by the release of vitamin C. Given that many compounds are known to exhibit color change with PDA vesicles, these compounds could be considered as potential candidate materials for future studies following this research. However, there are many factors to consider, such as solubility in water and selecting polymers that can be mixed with each other, so feasibility can only be determined through experimentation.

## 4. Conclusions

In this study, a new system that monitors vitamin C release through color change was prepared by electrospinning. Coaxial nozzles were used in electrospinning to prepare fibers with core shell structures. The core was composed of PEO incorporating vitamin C, and the shell was composed of PCL containing polydiacetylene as the indicator, which underwent color change upon contact with vitamin C. Polydiacetylene was synthesized using PCDA derivatives, in which the end functional group of PCDA was changed to an amine group. The obtained polydiacetylene exhibited a color change from blue to red upon direct contact with vitamin C. Similarly, the release experiments using fiber mats prepared using coaxial nozzles revealed a color change from blue to red upon vitamin C release.

## Figures and Tables

**Table 1:** Specifications of the electrospinning nozzle.

Type of Nozzle	Position	Gauge (G)	Diameter (mm)	
			ID	OD
DC	core	25	0.26	0.52
	shell	18	0.92	1.28

ID: inner diameter; OD: outer diameter.

**Table 2:** Electrospinning conditions * and average diameter of the formed fiber.

	Flow Rate (Shell)	Average Diameter
A	1.5 mL/h	3.9 ± 0.6 μm
B	2.0 mL/h	4.8 ± 0.6 μm
C	2.5 mL/h	5.7 ± 0.7 μm

* Flow rate (core) 0.50 mL/h.

## Data Availability

Data are contained within the article.
